# Osteochondroma as a Cause of Ischiofemoral Impingement – First Case Series

**DOI:** 10.15388/Amed.2021.28.1.15

**Published:** 2021-04-06

**Authors:** Bünyamin Güney, Emrah Doğan, Murat Yunus Özdemir

**Affiliations:** Muğla Sıtkı Koçman University, Faculty of Medicine, Radiology, Turkey; Muğla Sıtkı Koçman University, Faculty of Medicine, Radiology, Turkey; Muğla Sıtkı Koçman University, Faculty of Medicine, Radiology, Turkey

**Keywords:** Ischiofemoral impingement, Osteochondroma, Magnetic resonance, Quadratus femoris muscle impingement

## Abstract

Ischiofemoral impingement (*ISFI*)**is the compression of the *quadratus femoris *muscle resulting from the narrowed distance between the lesser trochanter and the ischial bone. Congenital factors (such as developmental hip dysplasia), positional conditions (such as femoral anteversion), intertrochanteric fractures, osteotomy, and osteoarthritis may lead to the superior and medial displacement of the femur which is causing the *ISFI*. According to the literature, osteochondroma (*OC*) is not described among the main etiological factors of *ISFI.* There is only one case report about the relationship between *ISFI *and *OC*. We present two *ISFI* cases due to *OC *accompanied by radiological findings. Our patients are 19 and 32 years old. Our article is the first case series on this topic.

## Abstract (lt)

**Osteochondroma – ischiofemoralinio ankštumo priežastis. Pirmoji klinikinių atvejų serija:**

**Santrauka. **Ischiofemoralinis ankštumas (ISFI) yra kvadratinio šlaunies raumens suspaudimas, atsirandantis dėl susiaurėjimo tarp mažojo gūbrio ir sėdynkaulio. Įgimti veiksniai (pvz., klubo sąnario vystymosi displazija), pozicinės sąlygos (pvz., femoralinė anteversija), intergūbriniai lūžiai, osteotomija ir osteoartritas gali išprovokuoti ISFI, sukeltą šlaunikaulio poslinkio į viršų ar centrą. Remiantis literatūra, osteochondroma (OC) nėra paminėta tarp pagrindinių ISFI etiologinių veiksnių. Žinomas tik vienas atvejis, kai nustatytas ISFI ir OC ryšys. Pateikiame du ISFI atvejus dėl OC kartu su radiologinėmis išvadomis. Mūsų pacientai yra 19 ir 32 metų amžiaus. Šis mūsų darbas yra pirmoji klinikinių atvejų serija šia tema.

**Raktažodžiai:** ischiofemoralinis ankštumas, osteochondroma, magnetinis rezonansas, kvadratinio šlaunies raumens suspaudimas

## Introduction

Hip pain is one of the common clinical symptoms in the elder population affecting 14.3% of patients over 60 years old. It is often associated with arthrosis. More specific causes should be considered when hip pain is detected in the young population. Ischiofemoral impingement syndrome (*ISFI*) is a cause of early hip pain that primarily affects middle-aged women. The impingement of the *quadratus femoris* muscle (*QFM*) resulting from the narrowed distance between the lesser trochanter and the ischial bone, is defined as *ISFI*. Congenital factors (such as developmental hip dysplasia), positional conditions (such as femoral anteversion), intertrochanteric fractures, osteotomy, and osteoarthritis may lead to the superior and medial displacement of the femur which is causing the ISFI [1]. According to the literature, osteochondroma (*OC*) is not among the main etiological factors of *ISFI*. Our article is the first case series on this topic.

## Cases:

### Case 1 

**Figure 1.**
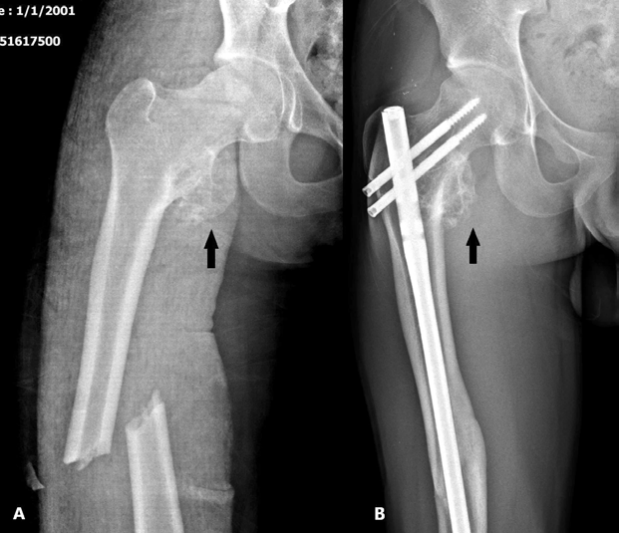
The dislocated fracture line in the right femoral diaphysis on AP X-ray radiography of a 19-year-old male case (A). Six months after the treatment, a healed fracture line and metallic operation material are observed (B). *Osteochondroma* localized at the level of the right *lesser trochanter* (shown with black arrows) on both X-rays.

A 19-year-old male patient was admitted to the emergency department after a traffic accident. X-ray radiography showed a displaced fracture line in the mid-diaphyseal part of the femur. There was also an exophytic bone lesion in the lesser trochanter. Primarily, the femur fracture was operated on. The patient was discharged after the operation. The control was suggested. He suffered pain intensified in the inner part of the femoral region. The pain increased when the leg was turned outward. The fracture line healed, and the callus formation was present in the X-ray (Figure 1).

**Figure 2.**
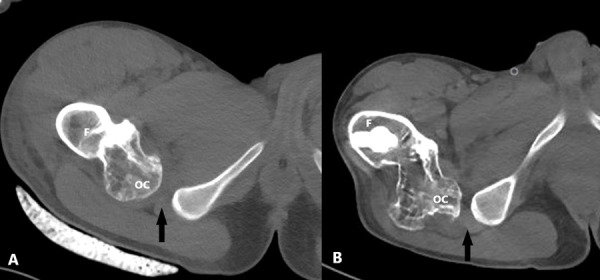
The axial hip CT image taken after trauma in the emergency department (before the operation) (A) and control axial CT image after six months from the operation (B). After the treatment, the *ischiofemoral distance* narrowed and the compression of the *quadratus femoris muscle* increased on CT (*ischiofemoral distance* indicated by black arrows). *F: Femur, OC: Osteochondroma*.

The pain was excessively severe for a noncomplicated healed fracture. CT scan was requested because of the exophytic bone lesion which was seen also in the first X-ray. On CT, a 5 cm diameter bone lesion continuing with normal bone cortex was observed. The mushroom-shaped head of the lesion was the demonstrative sign for *OC*. There was a severe impingement on the right *QFM *due to OC’s compression. Ischiofemoral (*IF*) distance was obliterated (Figure 2). 

**Figure 3.**
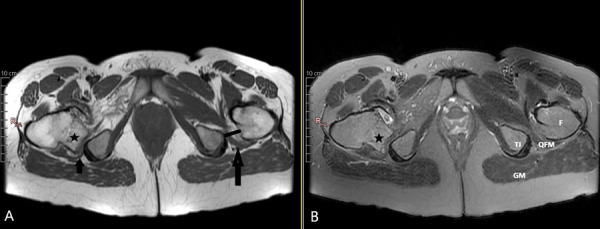
Hip MRI examination of the same patient. Fat saturation FSE T2W axial image (A) and FSE T1W axial image (B). *Osteochondroma* (asterisk) with a 6 mm cartilage cap that causes severe QFM compression by narrowing the right *ischiofemoral distance*. Normal *ischiofemoral distance* and QFM on the left. *GM: Gluteus maximus, TI: Tuber ischium, QFM: Quadratus femoris muscle, F: Femur.*

MRI was requested. There was a 5 mm thick cartilage cap of the lesion and *OC* diagnosis is proven. The lesion is narrowing the *IF *distance. Muscular edema was present due to a significant compression of the *QFM*. In comparison of old and new images, it was observed that the *IF* distance was narrower before fracture surgery and increased significantly after fracture reduction and healing (Figure 3).

The patient with the diagnosis of *ISFI* was referred to the orthopedic department for treatment with radiology reports.

### Case 2

**Figure 4.**
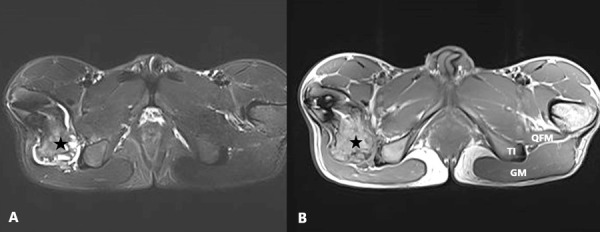
The hip MRI of the 32-year-old female patient. FSE T1W axial image (A) and fat saturation FSE T2W axial image (B). *Osteochondroma* (asterisk) causing severe *QFM* compression by narrowing the *ischiofemoral distance* on the right. Normal ischiofemoral distance and *QFM *(shown by black line and arrow) on the left. *GM: Gluteus maximus, TI: Tuber ischium, QFM: Quadratus femoris muscle, F: Femur.*

**Figure 5.**
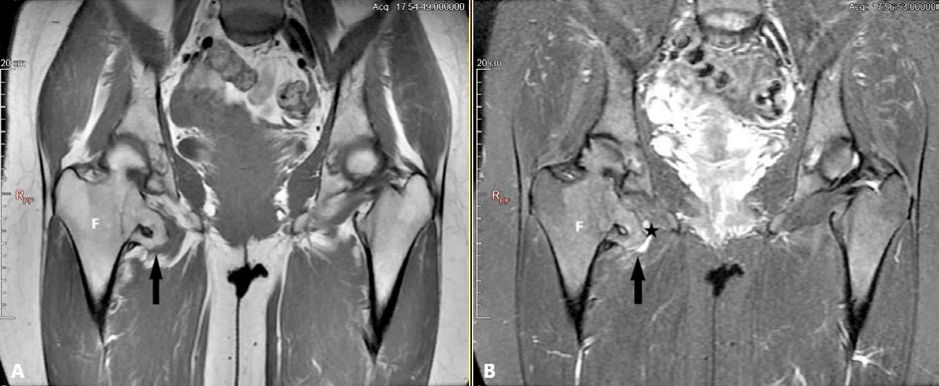
Coronal plane hip MRI images of the same patient. FSE T1W (A) and fat saturation FSE T2W (B). *Osteochondroma* (shown with black arrows) containing a 4 mm cartilage cap (asterisk) that creates *QFM* compression by narrowing the *ischiofemoral distance* on the right. Normal *ischiofemoral distance* on the left*. F: Femur.*

Our 32-year-old female patient was admitted to our outpatient clinic with severe pain in the medial femur and hip. In the anamnesis, our patient said that the pain increased with external rotation of the leg. She also reported that her pain was aggravated by stepping. The pain spread towards the inguinal zone and buttock. A popping sound was heard from the patient’s hip. It was an evidence of snapping hip resulting from impingement. There was also the movement restriction. In the X-ray graph, a lesion considered as *OC *was detected at the level of the lesser trochanter. MRI was requested. An *OC* of 32 mm size, extruded from the right lesser trochanter with 4 mm cartilage cap was seen. Severe compression on the right *QFM* by narrowing the *IF* distance was determined. The fat replacement resulting from atrophy was present (Figure 4-5).

The patient was diagnosed with* ISFI* resulting from *OC* and was referred to an orthopedic outpatient clinic.

## Discussion

*ISFI* was mentioned for the first time by Johnson in 1977. He showed in X-ray radiography that the distance between the lesser trochanter and *tuber ischiadicum* was narrow in three patients with persistent pain of the hip. Patients were completely improved after the resection of lesser trochanter [1,2]. Torriani e al. described *ISFI* in all aspects in 2009 [2].**

*ISFI* is a typical impingement*. QFM* is the most affected muscle. *QFM* takes this name because of the flat, quadrangular shape of the muscle. It is the most important external rotator muscle of the femur. It also helps adduction. *QFM* passes through the *IF *distance during its course and is close to the sciatic nerve at this distance [3]. The most important clinical complaints of the patients are pain in the hip and anterior femur. Pain may spread to the knee, buttock, and inguinal zones and increases with external rotation, adduction, and extension. The anamnesis for pain is not helpful in diagnosis when the pain scale is wide. The most important diagnostic physical examination is the anterior impingement test, and the finding is severe pain occurring in 90° of hip flexion, adduction, and internal rotation. This test is positive in 90% of the patients [4]. Both our patients have severe pain with external rotation of the leg.

*ISFI* was found in 10% to 40% of the random population in the studies performed by measuring the *IF *interval [5]. However, these high percentages probably don’t express the real patient population who need the treatment.

*ISFI* is a disease characterized by inflammation, muscular and perimuscular edema as well as bursitis in the acute phase, while fat replacement and decrease of muscle volume resulting from muscle compression are probable findings in the chronic phase.**The pain is especially in the medial part of the femur and inguinal part. The severity of the pain increases with hip extension, adduction, and external rotation. The slow onset of the disease makes it difficult to diagnose in daily practice. Magnetic resonance imaging (MRI) is the best method for the diagnosis of *ISFI* [1]. In our case series, the first patient was diagnosed incidentally after trauma. The second one was an outpatient. Although examination and other radiological methods gave valuable findings, they both were diagnosed with *ISFI *by MRI.

*OC* is one of the very rare causes of *ISFI*. If we browse PubMed with the following keywords; *‘Ischiofemoral impingement’* [All Fields] OR *‘quadratus femoris impingement*’ [All Fields] OR *‘quadratus femoris’*, 182 papers can be found in the last 5 years. Only one case report was about ISFI resulting from *OC* [6].

*OCs* are the most common benign bone tumors and constitute 10-15% of all bone tumors. They can be solitary or multiple [7]. The fractures and massive endocytosis’ may mimic *OC* [8]. *OC* is more common in males whereas *ISFI* in females. 75% of the *OCs *are encountered before the age of 20 [7]. They are mostly seen in the lower extremities, especially in the femur.* ISFI* is a disease of the middle age group.* OC* and *ISFI* age groups are different [9]. Probably this epidemiologic information is the factors that diminish the role of *OCs* as an etiology of *ISFI*.

X-ray findings are limited and not specific for *ISFI*. Patti et al. reported heterogeneity and sclerosis in the lesser trochanter and ischia as the most prominent X-ray findings. It can also give information about the narrowing of the distance between the *lesser trochanter* and the *ischium*. Ultrasonography studies have not shown a benefit in the diagnosis of *ISFI*. CT gives clear information about bone structures and narrowing of the *IF* interval, but it is insufficient to show soft tissue findings such as muscle edema and fat tissue replacement [10].

MRI is the best imaging method in the diagnosis of *ISFI*. In MRI, edema in the *QFM* is the most important finding suggesting *ISFI*. The lesser trochanter and tuber ischium are normally approximately 2 cm apart from each other. This distance allows the femur to rotate without touching the tuber ischium or hamstring tendon. *ISFI* is best evaluated on axial T2-weighted images. The presence of edema in the muscle instead of the absence of muscular fibers’ deterioration help to distinguish *IF *compression from *QFM* tear. In the chronic period, T1-weighted MRI images are useful in evaluating quadratus femoris atrophy, hyperintense fat replacement, and muscle volume loss associated with *ISFI *[11].

Initial treatment may be conservative therapy that includes rest, activity restriction, anti-inflammatory nonsteroidal drugs, percutaneous ultrasound therapy, and physical therapy. CT-guided or ultrasound-guided injections with anesthetics and steroids can be used as a diagnostic test and for a symptomatic relief [12]. Surgical treatment options include resection of the lesser trochanter and decompression of the *QFM* [13].

## Conclusion

*ISFI* is one of the causes of hip pain in the early age group. This is the first case series about the relationship between *OC* and *ISFI*. The patient should be evaluated in terms of *ISFI*, especially in *OC*s located in the lesser trochanter or ischium pubis arm.
